# Supplementary material for: Direct detection of 4-dimensions of SARS-CoV-2: infection (vRNA), infectivity (antigen), binding antibody, and functional neutralizing antibody in saliva
Source: Sci Rep. 2024 Dec 28;14:30792. doi: 10.1038/s41598-024-81019-4 (PMC11681011; doi:10.1038/s41598-024-81019-4)
Supplement: Supplementary file 1 — Supplementary Material 1 [file 41598_2024_81019_MOESM1_ESM.docx]

Direct Detection of 4-Dimensions of SARS-CoV-2: Infection (vRNA), Infectivity (Antigen), Binding Antibody, and Functional Neutralizing Antibody in Saliva

Aida Mohammadi^1^, Samantha Chiang^1^, Feng Li^1^, Fang Wei^1^, Chang S. Lau^2^, Mohammad Aziz^1^, Francisco J. Ibarrondo^3^, Jennifer A. Fulcher^3^, Otto O. Yang^3^, David Chia^1^, Yong Kim^*1^ and David T.W. Wong^*1^

^1^School of Dentistry, University of California Los Angeles, Los Angeles, CA, USA

^2^GenScript USA Inc.

^3^Division of Infectious Diseases, Department of Medicine, David Geffen School of Medicine, University of California Los Angeles, Los Angeles, CA, USA

Supplementary Table 1: COVID status and vaccination data of outpatient COVID-19 patient cohort in BAb assay

| Number | Days since onset of symptoms | Type of vaccination | Days since 1st vaccination | Days since 2nd vaccination |
| --- | --- | --- | --- | --- |
| 1 | 330 | Moderna | 54 | 28 |
| 2 | 338 | Moderna | 11 |  |
| 3 | 330 | Moderna | 54 | 28 |
| 4 | 98 | Moderna | 14 |  |
| 5 | 341 | Moderna | 8 |  |
| 6 | 316 | Pfizer | 14 |  |
| 7 | 359 | Pfizer | 29 | 8 |
| 8 | 301 | Pfizer | 21 |  |
| 9 | 313 | Pfizer | 29 |  |
| 10 | 86 | Moderna | 17 |  |
| 11 | 53 | Moderna | 18 |  |
| 12 | 52 | Moderna | 28 |  |
| 13 | 146 | Moderna | 45 | 17 |

Supplementary Table 2: COVID status and vaccination data of outpatient COVID-19 patient cohort in NAb assay

| Number | Days since onset of symptoms | Type of vaccination | Days since 1st vaccination | Days since 2nd vaccination |
| --- | --- | --- | --- | --- |
| 1 | 338 | Moderna | 11 |  |
| 2 | 330 | Moderna | 54 | 28 |
| 3 | 345 | Pfizer | 15 |  |
| 4 | 87 | Moderna | 3 |  |
| 5 | 98 | Moderna | 14 |  |
| 6 | 330 | Moderna | 54 | 28 |
| 7 | 360 | Moderna | 33 | 5 |
| 8 | 341 | Moderna | 8 |  |
| 9 | 316 | Pfizer | 14 |  |
| 10 | 331 | Pfizer | 29 | 7 |
| 11 | 344 | Pfizer | 42 | 20 |
| 12 | 338 | Pfizer | 8 |  |
| 13 | 359 | Pfizer | 29 | 8 |
| 14 | 301 | Pfizer | 21 | 0 |
| 15 | 126 | Moderna | 42 | 12 |
| 16 | 86 | Moderna | 17 |  |
| 17 | 104 | Moderna | 35 | 10 |
| 18 | 53 | Moderna | 18 |  |
| 19 | 52 | Moderna | 28 |  |
| 20 | 327 | Moderna | 12 |  |
| 21 | 146 | Moderna | 45 | 17 |
| 22 | 442 | Pfizer | 43 | 19 |
| 23 | 465 | Moderna | 75 | 47 |
| 24 | 299 | Pfizer | 54 | 33 |

Supplementary Table 3: Vaccination data of the vaccinated infection naïve patient cohort in BAb assay

| Number | Type of vaccination | Days since 1st vaccination | Days since 2nd vaccination |
| --- | --- | --- | --- |
| 1 | Pfizer | 29 | 7 |
| 2 | Pfizer | 18 |  |
| 3 | Pfizer | 71 | 47 |
| 4 | Pfizer | 32 | 11 |
| 5 | Pfizer | 32 | 11 |
| 6 | Pfizer | 73 | 52 |
| 7 | Pfizer | 33 | 12 |
| 8 | Pfizer | 42 | 21 |
| 9 | Pfizer | 21 |  |
| 10 | Pfizer | 47 | 15 |
| 11 | Moderna | 41 | 12 |
| 12 | Moderna | 13 |  |
| 13 | Pfizer | 44 | 21 |

Supplementary Table 4: Vaccination data of the vaccinated infection naïve patient cohort in NAb assay

| Number | Type of vaccination | Days since 1st vaccination | Days since 2nd vaccination |
| --- | --- | --- | --- |
| 1 | Pfizer | 32 | 11 |
| 2 | Pfizer | 32 | 11 |
| 3 | Pfizer | 45 | 24 |
| 4 | Pfizer | 33 | 12 |
| 5 | Pfizer | 42 | 21 |
| 6 | Moderna | 13 |  |
| 7 | Pfizer | 44 | 21 |

Supplementary Table 5. The sequences of capture and detect probes

| Primer set | Sequence |
| --- | --- |
| *N2 set* |  |
| F3 | ACCAGGAACTAATCAGACAAG |
| B3 | GACTTGATCTTTGAAATTTGGATCT |
| FIB | TTCCGAAGAACGCTGAAGCG GAACTGATTACAAACATTGGCC |
| BIP | CGCATTGGCATGGAAGTCA CAATTTGATGGCACCTGTGTA |
| LF | GGGGGCAAATTGTGCAATTTG |
| LB | CTTCGGGAACGTGGTTGACC |
| *NL set* |  |
| F3 | TTGCTGAATAAGCATATTGACG |
| B33 | TGAGTTTAGGCCTGAGTTGAG |
| FIB | GTCTCTGCGGTAAGGCTTGAG ATACAAAACATTCCCACCAACA |
| BIP | GCAAACTGTGACTCTTCTTCCTGC GCACTGCTCATGGATTGTTG |
| LF | TCATCAGCCTTCTTCTTTTTGTCCT |
| LB | GCAGATTTGGATGATTTCTCCAAAC |
| *EFIRM probes* |  |
| N2 Capture probe | AAAAAAAAAAGAAATAAACAAATAAAACAATAACAAATAAAAAAAAACAAATA AACAATAAAAAAAAACAATGCCAATGCGCGACA |
| N2 Detect probe | TTCCGAAGAACGCTGAA |
| NL Capture probe | AAAAAAAAAAGAAATAAACAAATAAAACAATAACAAATAAAAAAAAA CAAATAAACAATAAAAAAAAACAAGTCACAGTTTGCTGT |
| NL Detect probe | TTCTTCTGTCTCTGCGG |

Supplementary Table 6. Comparison of EFIRM SARS-CoV-2 antigen assay with other EUA antigen assays

| EUA\Parameter | LOD (TCID₅₀) | Sensitivity | Specificity | Assay Results |
| --- | --- | --- | --- | --- |
| Sofia SARS Antigen Fluorescent Immunoassay (FIA) | 1.13 × 102 per mL  (NR-52286) | 96.7% | 100% | Qualitative |
| Veritor System for Rapid Detection of SARS-CoV-2 | 1.4 × 102 per mL  (NR-52287)? | 84% | 100% | Qualitative |
| LumiraDx SARS-CoV-2 Ag Test | 32 per mL  (NR-52287) | 97.6% | 96.6% | Qualitative |
| BinaxNOW COVID-19 Ag Card | 22.5 per swab | 97.1% | 98.5% | Qualitative |
| Sampinute™ COVID-19 Antigen MIA | 1.2 × 102 per mL  (Unknown biorepository)  Nasal swab | 94% | 100% | Qualitative |
| CareStart™ COVID-19 Antigen | 8 × 102 per mL  (NR-52286)  Nasal swab | 88.37% | 100% | Qualitative |
| EFIRM – N protein assay | 3. 5 per mL  (NR-52287) | 99.8% < 10 days | 100% | Quantitative |

Supplementary Table 7. PRNT50 result interpretation

| Value Result (Dilution Titer) | Result | Test Result Interpretation |
| --- | --- | --- |
| > 1:20 | Positive | Neutralizing antibodies for SARS-CoV-2 are detected at 50% viral neutralization. |
| ≤ 1:20 | Negative | Neutralizing antibodies for SARS-CoV-2 are not detected at 50% viral neutralization. |

| **a** | **b** |
| --- | --- |
| 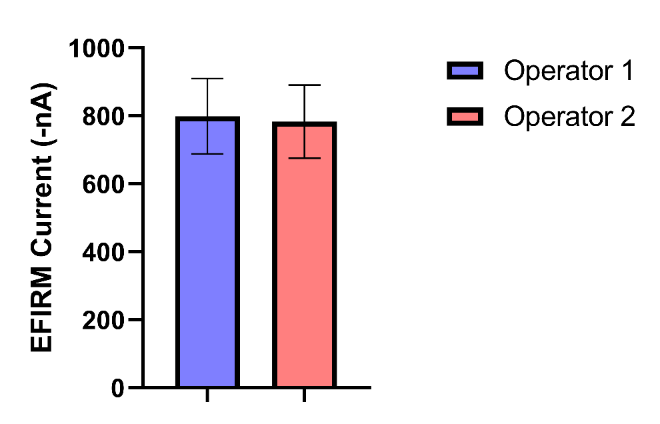 | 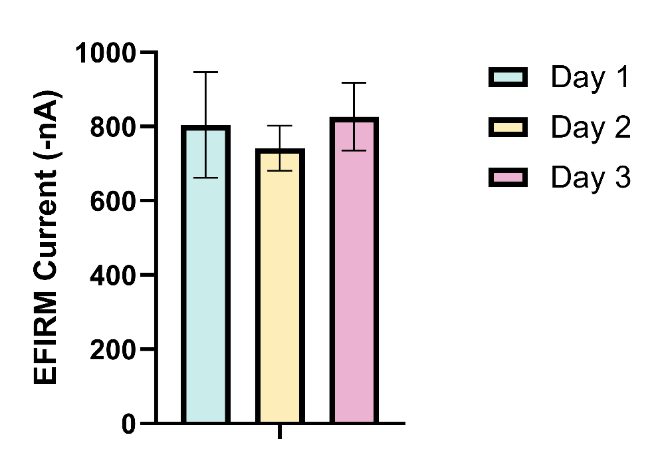 |
|  |  |
| **c** | **d** |
| 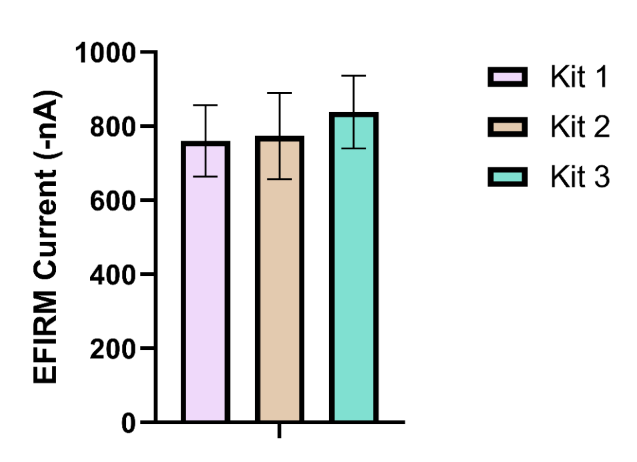 | 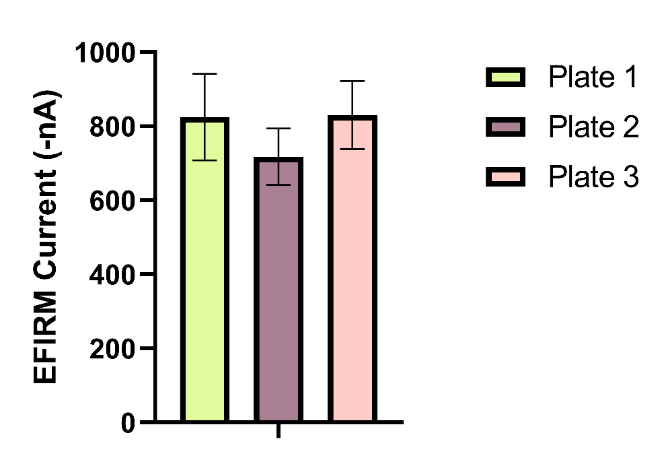 |

Supplementary Figure 1. The effect of different factors on the baseline EFIRM current of NAb assay (**a**) Effect of operators (**b**) Effect of days (**c**) Effect of kits (**d**) Effect of plates
